# β-casein nanovehicles for oral delivery of chemotherapeutic drug combinations overcoming P-glycoprotein-mediated multidrug resistance in human gastric cancer cells

**DOI:** 10.18632/oncotarget.8019

**Published:** 2016-03-10

**Authors:** Maya Bar-Zeev, Yehuda G. Assaraf, Yoav D. Livney

**Affiliations:** ^1^ Russell Berrie Nanotechnology Institute, Technion - Israel Institute of Technology, Haifa 3200000, Israel; ^2^ The Laboratory of Food Physical Chemistry and Biopolymeric Delivery Systems for Health, Department of Biotechnology and Food Engineering, Technion - Israel Institute of Technology, Haifa 3200000, Israel; ^3^ The Fred Wyszkowski Cancer Research Laboratory, Department of Biology, Technion - Israel Institute of Technology, Haifa 3200000, Israel

**Keywords:** β-casein micelles, target-activated oral delivery, gastric cancer, multidrug resistance reversal, paclitaxel-tariquidar combination

## Abstract

Multidrug resistance (MDR) is a primary obstacle to curative cancer therapy. We have previously demonstrated that β-casein (β-CN) micelles (β-CM) can serve as nanovehicles for oral delivery and target-activated release of hydrophobic drugs in the stomach. Herein we introduce a novel nanosystem based on β-CM, to orally deliver a synergistic combination of a chemotherapeutic drug (Paclitaxel) and a P-glycoprotein-specific transport inhibitor (Tariquidar) individually encapsulated within β-CM, for overcoming MDR in gastric cancer. Light microscopy, dynamic light scattering and zeta potential analyses revealed solubilization of these drugs by β-CN, suppressing drug crystallization. Spectrophotometry demonstrated high loading capacity and good encapsulation efficiency, whereas spectrofluorometry revealed high affinity of these drugs to β-CN. *In vitro* cytotoxicity assays exhibited remarkable synergistic efficacy against human MDR gastric carcinoma cells with P-glycoprotein overexpression. Oral delivery of β-CN - based nanovehicles carrying synergistic drug combinations to the stomach constitutes a novel efficacious therapeutic system that may overcome MDR in gastric cancer.

## INTRODUCTION

Gastrointestinal cancers are the third leading cause of cancer-related mortality worldwide [1]. Gastric cancer is classified into two histologically distinct types: intestinal and diffuse. Intestinal-type tumor cells have tubular structures characterized by reduced stroma that frequently ulcerate providing a barrier-free path for drugs to infiltrate the tumor from the gastric lumen, whereas, the diffuse-type tumors cause mucosal dysplasia [2].

Most current chemotherapeutics are administered intravenously (IV), causing discomfort and stress to patients, and high costs due to multiple hospitalizations required to complete a series of IV chemotherapeutic sessions [3, 4]. Furthermore, hospitalization is dangerous for immunocompromised cancer patients, due to drug-resistant pathogens widespread in hospitals [5]. Moreover, many patients who apparently respond to first-line chemotherapy frequently face tumor progression or recurrence, necessitating additional chemotherapeutic cycles. Consequently, the response decreases and the therapy becomes ineffective due to the frequent emergence of multiple drug resistance phenomena [6–10].

Multidrug resistance (MDR) is a major hindrance to curative cancer therapy [6]. Drug resistance frequently occurs as a primary (inherent) or secondary (acquired following chemotherapeutic treatment) tumor defense mechanism against multiple cytotoxic drugs. One of the major mechanisms of MDR is enhanced energy-dependent efflux of numerous hydrophobic cytotoxic drugs, which diffuse into cells through the plasma membrane [11]. This ATP-dependent efflux is mediated by transmembrane transporters of the ATP-binding cassette (ABC) superfamily, of which P-glycoprotein (P-gp/ABCB1) is considered the most dominant [11]. P-gp is overexpressed in a variety of carcinomas [12] where it markedly suppresses the efficacy of numerous hydrophobic chemotherapeutics. Much research focused on the development of compounds, known as chemosensitizers, that inhibit MDR efflux transporters, to achieve MDR reversal and re-sensitization of MDR cancer cells to chemotherapy [11, 13].

Oral drug administration is a highly preferred drug delivery route, promoting patient satisfaction and compliance [4, 14]. It does not require medical equipment or assistance, and thereby does not necessitate hospitalization. The availability of suitable and effective oral therapeutics would make a significant contribution to patients' quality of life, allowing less painful treatment at the comfort of their home, circumvent infection with antibiotic-resistant pathogens and significantly reduce costs [15, 16]. However, many drugs cannot be administered orally due to diverse factors, e.g. poor water solubility, limiting their oral bioavailability and hampering their pharmacokinetic and pharmacodynamic profiles [14–16].

Bovine β-casein (β-CN), one of the major proteins in milk, has a prominent amphiphilic structure [17] enabling its self-assembly into stable micellar structures in aqueous solution [18]. We have recently demonstrated the potential of β-CN micelles (β-CM) as nanovehicles for oral delivery of hydrophobic cargo (including bioactives and chemotherapeutics [3, 19–21]) facilitating encapsulation, stabilization [3, 19, 20, 22], protection, and target-activated release under simulated gastric digestion [21]. We have also shown that casein micelles enable encapsulation of hydrophobic bioactives [23] and promote their bioavailability, in a human clinical study [24]. β-CN remains intact in saliva [25], however upon simulated gastric digestion it rapidly degrades [21, 25]. Hence, β-CM may serve as an efficient oral delivery nanovehicle for target-activated release of chemotherapeutic drugs for local treatment of gastric cancer, thereby minimizing toxic side effects caused by systemic chemotherapy. Drug entrapment in a gastrically-digestible protein shell diminishes untoward toxicity to the upper gastro-intestinal tract (buccal cavity and esophagus). Minimal toxicity is expected for the healthy stomach tissue because of the thick mucosal layer, which is depleted in the tumor area of intestinal-type gastric tumors [2]. The β-CM delivery system is not intended to provide cancer-cell-specific targeting, but rather target-activated release in the stomach. However, it may be expected to obtain an improved tumor-focused local treatment in the case where the gastric tumor forms a local discontinuity in the mucosal lining of the internal stomach surface, or in the case of post-surgical chemotherapy.

Paclitaxel (PTX) is a highly lipophilic [26] chemotherapeutic drug (log *P* = 4.54 [27]), hence aqueous solubilization or encapsulation is required for its administration. PTX inhibits mitosis by binding to β-tubulin, leading to the formation of stable, non-functional, microtubules hence blocking cellular proliferation [28]. As most chemotherapeutic regimens, PTX causes serious side effects, including peripheral nervous system toxicity [27], due to insufficient selectivity. Its limited tumor-bioaccessibility leads to large doses, inducing higher untoward toxicity and emergence of MDR; PTX is a *bona fide* P-gp transport substrate [29]. Tariquidar (TQD) (log *P* = 6.1) [30] is a potent, third-generation P-gp transport inhibitor, and thus a specific MDR chemosensitizer. TQD acts either by avid noncompetitive binding to P-gp, or by inhibiting P-gp ATPase activity, or both [31].

Herein we developed a novel β-CN-based target-activated nanosystem for oral delivery of synergistic combinations of hydrophobic chemotherapeutic drugs and MDR chemosensitizers. We designed this nanosystem for treatment of MDR gastric cancer, exploiting both the ability of β-CN to self-assemble into micelles (while entrapping hydrophobic drugs) and its evolutionary ability to be easily digested in the stomach. Because MDR chemosensitizers enhance both bioavailability [16, 32] and cytotoxic activity [33, 34] of chemotherapeutics in cancer cells, we expect that such pairs of encapsulated drugs would display synergy in overcoming MDR gastric cancers. It is possible to co-encapsulate drug combinations within the same nanoparticle, or to individually encapsulate each drug, and later prepare the desired combinations by mixing the encapsulated drugs. By pre-encapsulating each drug separately we may form modular powder forms of the individually encapsulated drugs, which could then be easily mixed by the pharmacist according to the intended personalized treatment, without having to encapsulate the specific mixture. Furthermore, individual drug encapsulation enables avoiding drug-drug interactions thus extending product self-life. Therefore, the primary goal of the current research was to individually encapsulate a chemotherapeutic drug (PTX) and a chemosensitizer (TQD) within β-CM, and to evaluate the efficacy of their combined system against human P-gp-dependent MDR gastric cancer cells.

## RESULTS

### Drug-β-CN binding studies

**Figure 1:**
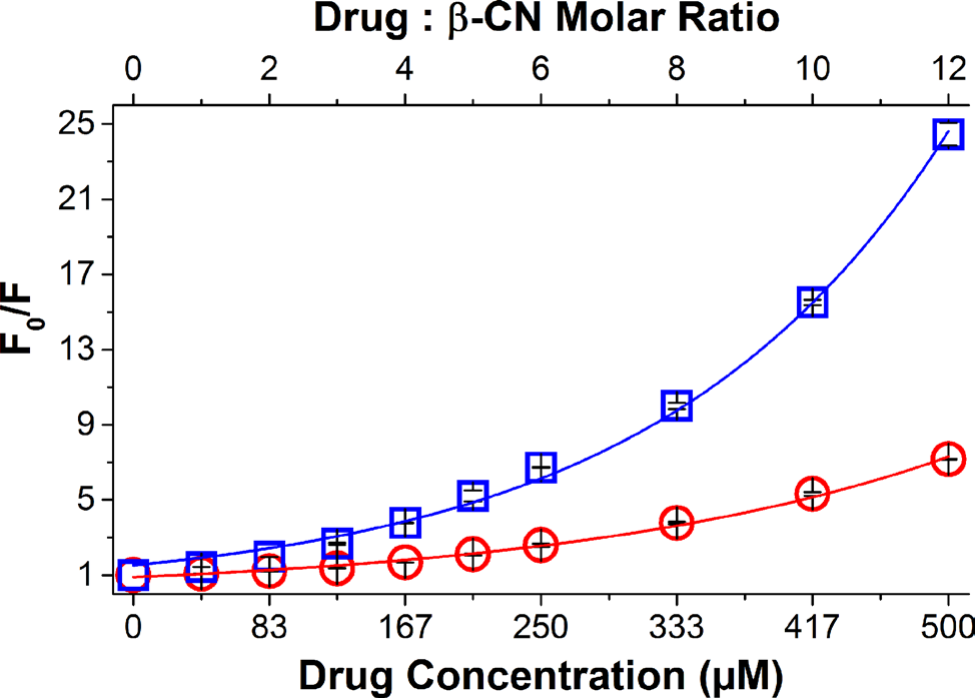
Drug-β-CN binding studies Modified Stern-Volmer plots for β-CN Trp143 fluorescence quenching by PTX (○) and TQD (□) as a function of drug:β-CN molar ratio (top axis) and drug concentration (bottom axis). Excitation: 270 nm and Emission: 356 nm. Lines represent model fits; error bars represent standard error (SE).

The comparison between the absorbance curve of the β-CM and the summation curve of their components along with the photograph of the pure drugs vs. drug-β-CM (Supplementary Figure S1), provided qualitative evidence that drug binding to β-CN occurred. This also confirmed the existence of binding-induced static quenching, supporting further quantitative study of drug binding to β-CN using fluorescence (Ex. 270 nm; Em. 356 nm) [35]. Figure 1 shows the increase in fluorescence intensity ratio of β-CN and drug-loaded β-CM as a function of drug:β-CN molar ratio. The association constant (K_SV_) and the static quenching constant (V) calculated by non-linear curve fitting (Equation (2)) [36] are presented in Table 1. Evidently, both PTX and TQD bind to β-CN; about 85% and 95% of the initial emission intensity of Trp143 was quenched by PTX and TQD, respectively. The association constant of PTX-β-CN (Table 1) is in accord with that obtained previously in our lab [19]. Thus, β-CN exhibits a 2-fold higher affinity for TQD than for PTX.

**Table 1:** Association constants, static quenching constants and maximal loading capacities of PTX-β-CM and TQD-β-CM

	K_SV_ ± SE (10^4^ M^−1^)	V ± SE (10^4^ M^−1^)	L_max_ ± SE (mg drug/g βCN)
PTX-β-CM	0.96 ± 0.07^a^	0.42 ± 0.01^a^	175 ± 20^a^
TQD-β-CM	1.80 ± 0.16^a^	0.56 ± 0.01^a^	136 ± 14^b^

a R^2^ = 0.98

b R^2^ = 0.99

Values presented are means ± SE.

**Figure 2:**
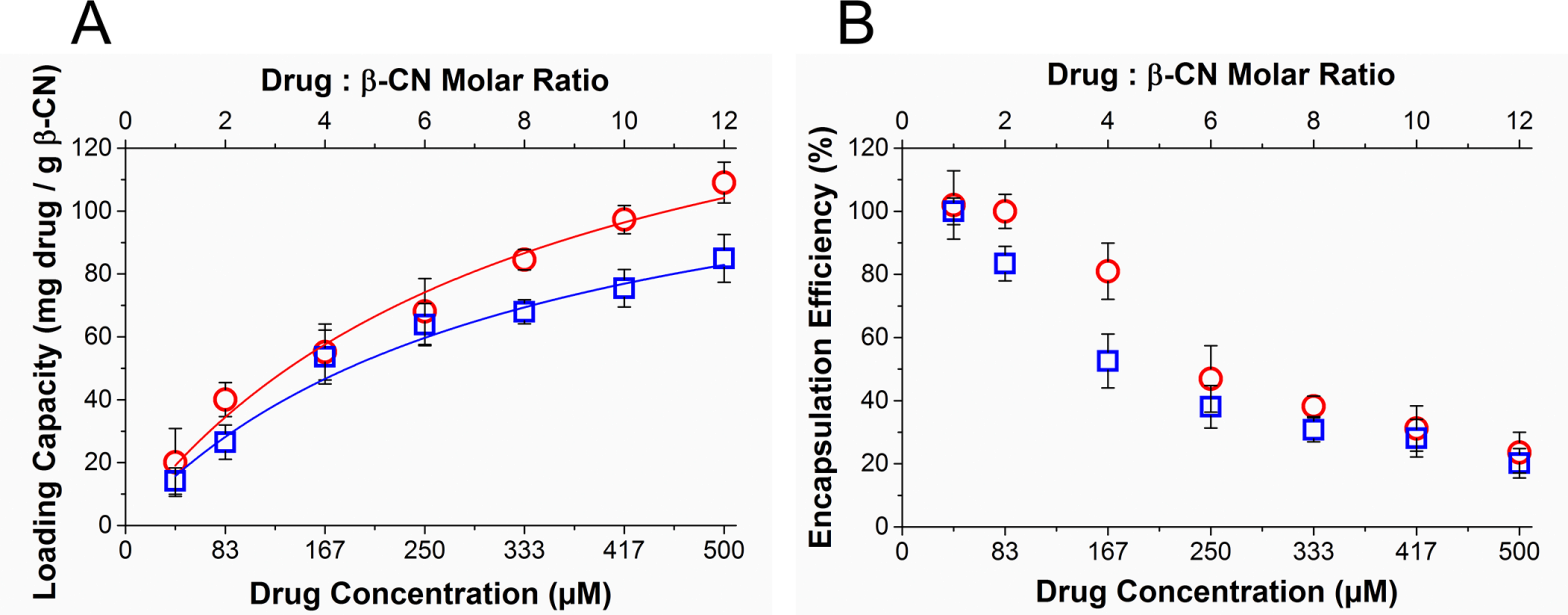
Drug loading capacity and encapsulation efficiency (**A**) Loading capacity and (**B**) encapsulation efficiency of PTX (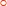) and TQD (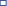) as a function of drug:β-CN molar ratio (top axis) and drug concentration (bottom axis). Lines represent Langmuir model curve fits; error bars represent SE.

Loading capacity and encapsulation efficiency of each of the drugs were determined by centrifugal sedimentation of unbound excess drug at increasing drug:β-CN molar ratios (Figure 2). Maximal loading capacities (L_max_) of PTX and TQD in β-CM, calculated from the asymptote of the Langmuir model curve fit (Equation (5)), are presented in Table 1. Expectedly, increasing drug concentration led to increasing drug loading capacity and decreasing encapsulation efficiency.

### Drug-β-CN binding stoichiometry

**Figure 3:**
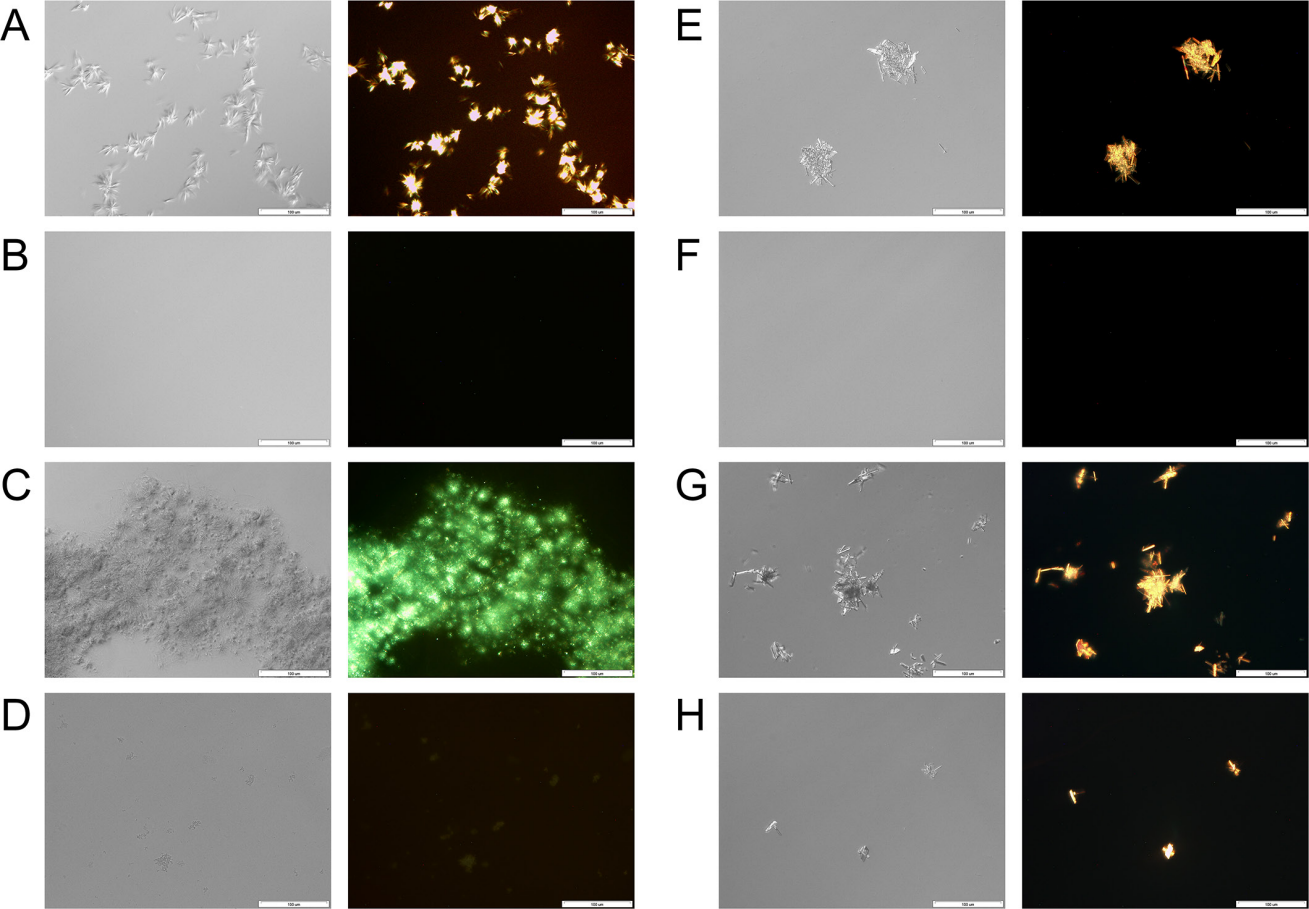
Drug-β-CN morphology and crystallinity visualization Nomarski DIC (left) and polarized light (right) microscopy images (×20 magnification) of: (**A**) 667 μM, (**C**) 833 μM pure PTX in PBS vs. (**B**) 667 μM, (**D**) 833 μM PTX in 1 mg/ml β-CN (16:1, 20:1 drug:β-CN molar ratio, respectively) and (**E**) 208 μM, (**G**) 333 μM pure TQD in PBS vs. (**F**) 208 μM, (**H**) 333 μM TQD in 1 mg/ml β-CN (5:1, 8:1 drug:β-CN molar ratio, respectively). Scale bar: 100 μm.

A comparison between light microscopy images of pure PTX or TQD in PBS and those in the presence of β-CN (Figure 3) confirms that drug concentrations were above the solubility limit in aqueous solution, forming micrometer-sized crystal aggregates, while in the presence of β-CN, sub-micron particles were formed. Apparently, β-CN suppressed drug crystal growth and aggregation via efficient encapsulation. Maximal loading capacity was observed at 5:1 TQD:β-CN molar ratio (F), while above it, TQD crystals were observed (H). 20:1 PTX:β-CN molar ratio revealed PTX-β-CM aggregates that modestly glow under polarized light (D). Super-saturation of PTX-β-CM was unattainable due to the limitation of PTX stock solution solubility and restriction of DMSO volume percentage in the samples. This result (D) suggested that the maximal loading capacity is about 20:1 PTX:β-CN molar ratio, based on the minute drug aggregates observed.

### Analysis of nanoparticle size distribution by DLS

**Figure 4:**
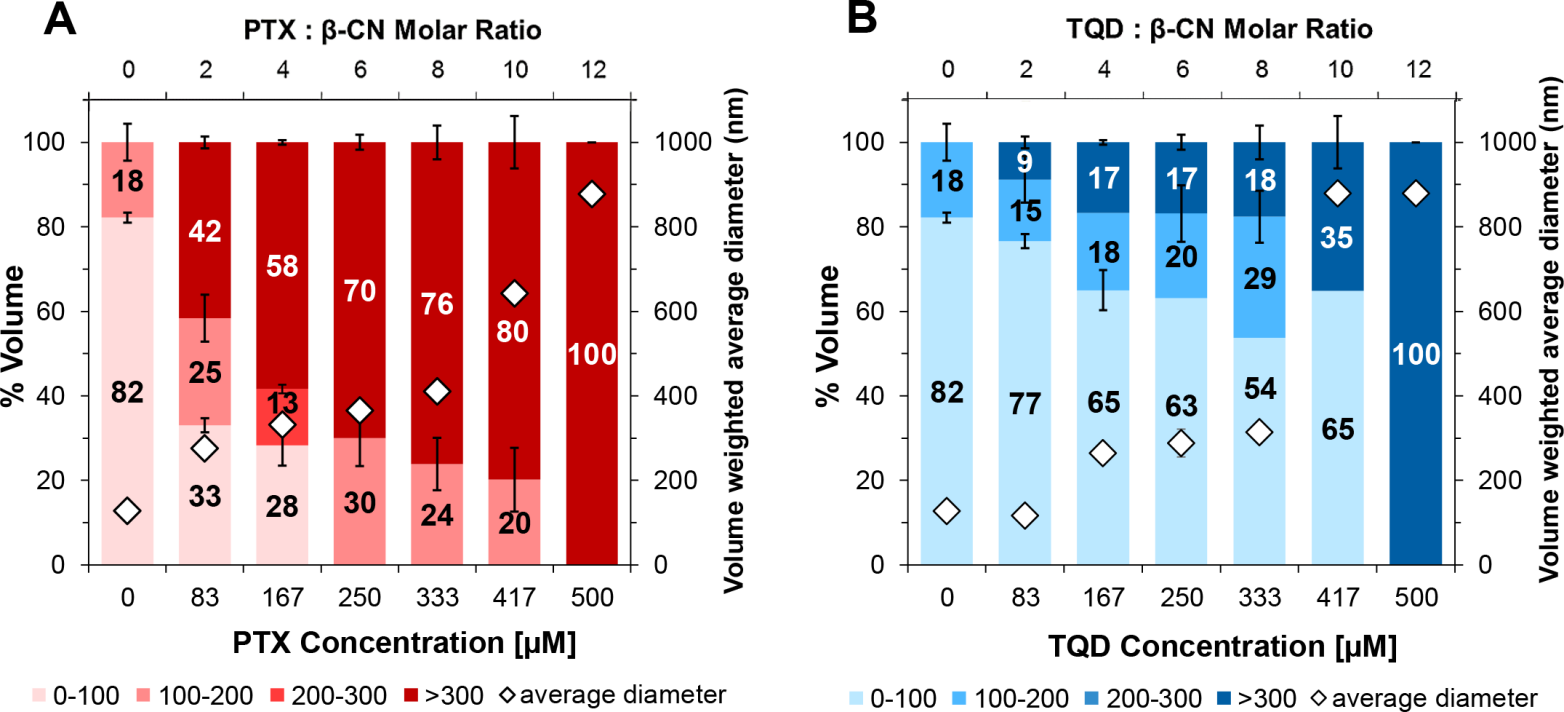
Nanoparticle size distribution Volume-weighted percent of (**A**) PTX and (**B**) TQD in 1 mg/ml β-CM and overall average diameter (◊) as a function of the molar ratio of drug:β-CN (top axis) and drug concentration (bottom axis); error bars represent SE.

DLS was used to evaluate the size distribution of PTX and TQD entrapped in β-CN at increasing drug:β-CN molar ratios. Figure 4 reveals that particle size distribution depends on the drug:β-CN molar ratio. At low TQD concentrations, over 70% of the particles were < 100 nm. Upon increase in the molar ratio, TQD apparently binds first to the hydrophobic core of the β-CM. When the micellar particles are maximally loaded with TQD, excess of the drug starts forming larger aggregates, appearing as a sub-population of larger particles that grow into microcrystals at very high TQD:β-CN molar ratios. This process has been previously identified by our group [19, 21]. PTX-β-CM display a similar structural organization including intermediate PTX-β-CM aggregates incorporating nano-sized PTX crystals. The results are supported by light microscopy images (Figure 3). These findings show that β-CN stabilizes PTX and TQD against crystallization and aggregation.

### Zeta potential analysis

**Figure 5:**
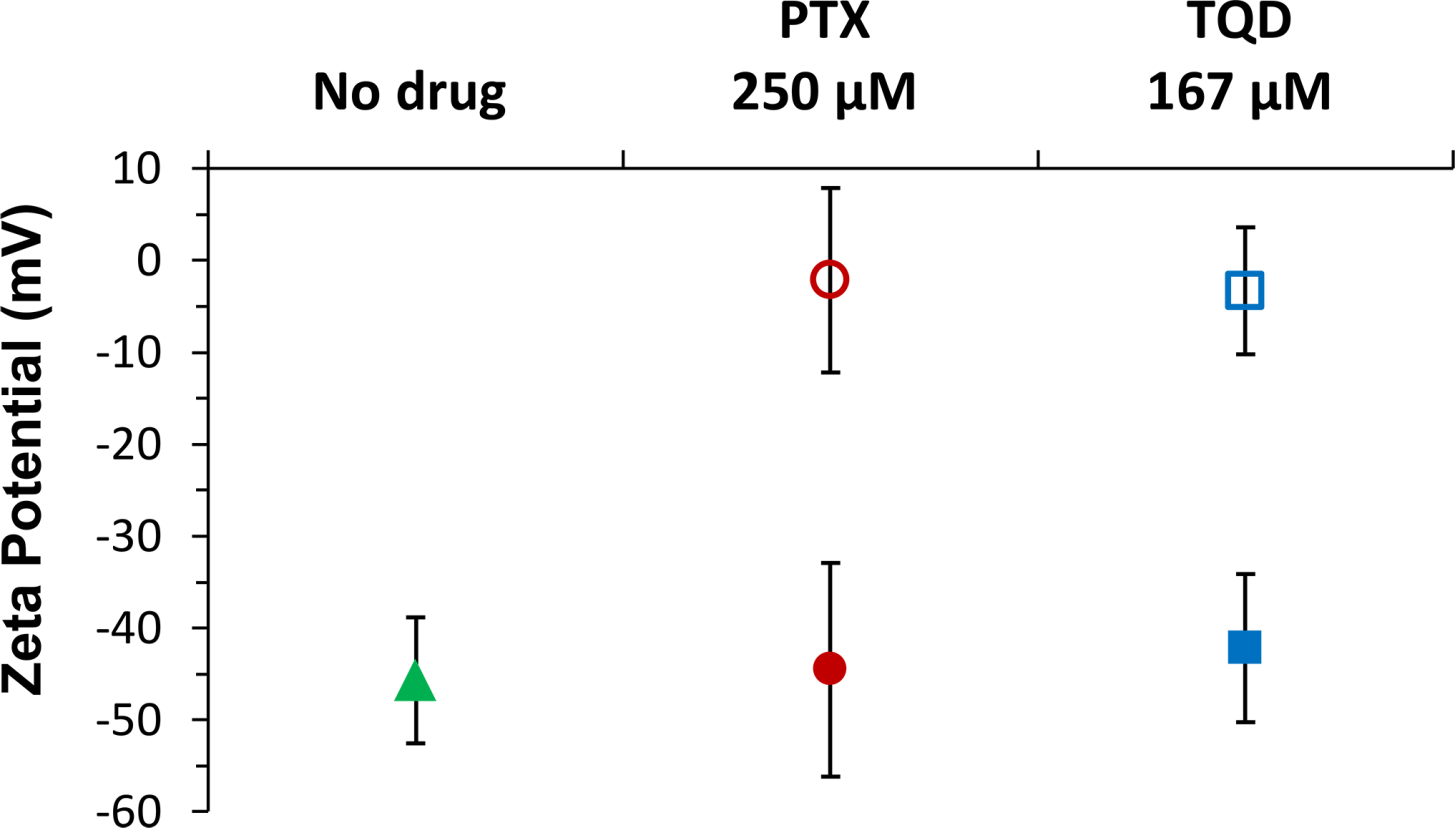
Zeta potential measurements Zeta potential of 1 mg/ml (42 μM) pure β-CN (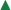), 250 μM PTX (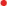) and 167 μM TQD (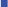) in 1 mg/ml β-CM (6:1 and 4:1 drug:β-CN molar ratio, respectively) for each of the drugs, at the same concentration, in PBS (corresponding empty symbols); error bars represent SE.

Zeta potential results of β-CN and pure drug solutions in PBS vs. solutions at the same drug concentrations of drug-β-CN (1 mg/ml β-CN, at 6:1 drug:β-CN molar ratio for PTX and 4:1 for TQD) are presented in Figure 5. β-CN in PBS showed zeta potential value of −45.6 mV. The zeta potentials of PTX and TQD in PBS were significantly closer to 0 mV suggesting a colloidally unstable system. However in the drug:β-CM molar ratios chosen for the *in vitro* cytotoxicity experiment both drugs in β-CM showed zeta potential values more negative than −40 mV, in line with our previous study [19], indicating a colloidally stable system.

### P-gp expression in human gastric carcinoma cell lines by Western blot analysis

**Figure 6:**
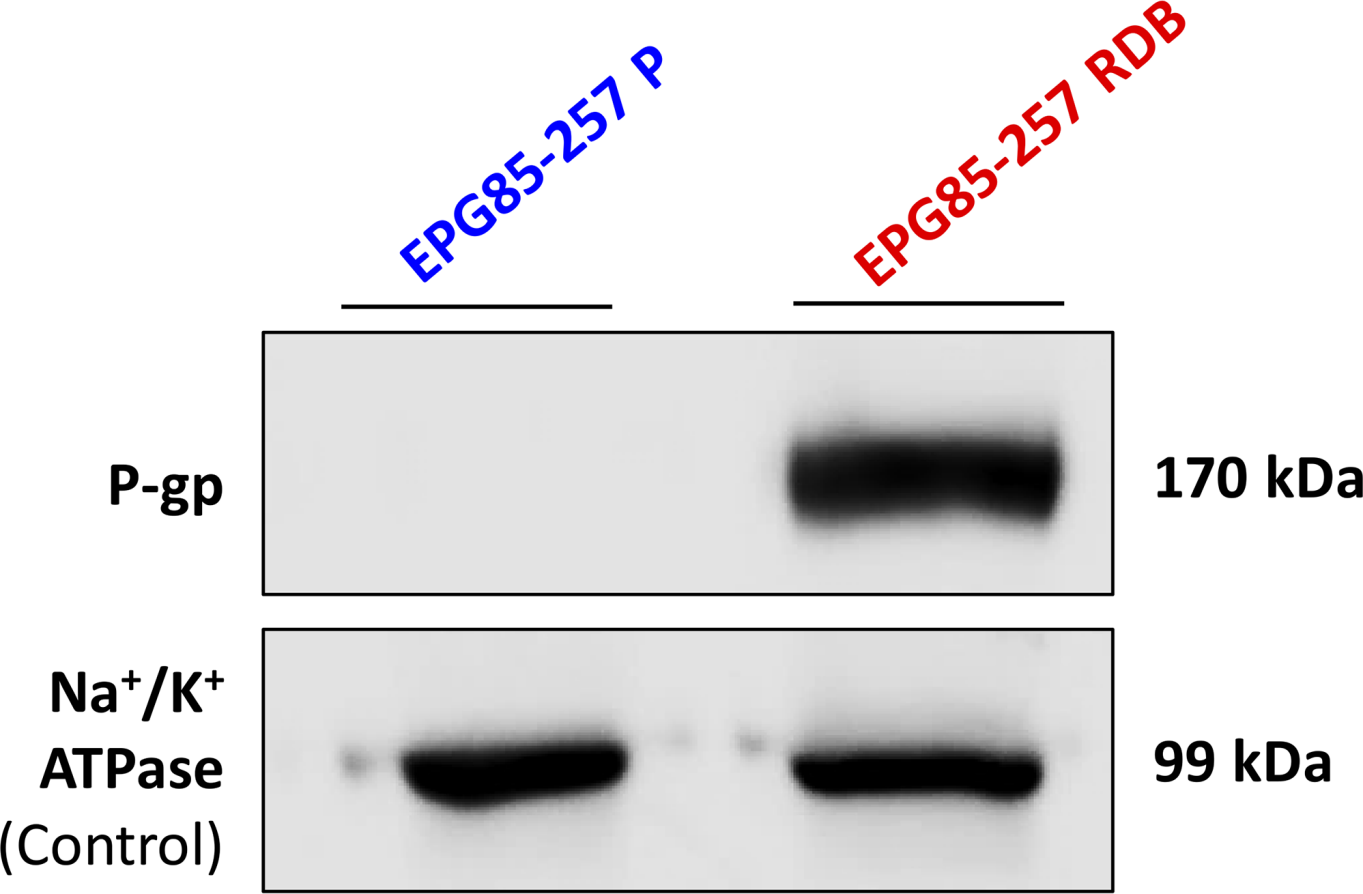
Quantification of P-gp expression in human gastric carcinoma cell lines Western blot analysis (10 μg protein/lane) was performed using JSB-1 monoclonal antibody directed against P-gp (170 kDa), to compare its expression in EPG85-257P cells and their MDR subline, EPG85-257RDB. Equal amounts of protein loading was confirmed using anti-KETTY antibody directed against the α–subunit of Na^+^/K^+^ ATPase (99 kDa). Protein concentration was determined by the Bradford protein assay.

Western blot analysis was used to quantify actual P-gp levels in gastric carcinoma cell lines (Figure 6). The MDR EPG85-257RDB subline overexpressed high levels of P-gp, whereas P-gp was not detectable in its parental EPG85-257P counterpart cells. Equal protein loading was verified using an antibody against the α-subunit of Na^+^/K^+^ATPase.

### Evaluation of the cytotoxic activity of drug-loaded β-CM

**Figure 7:**
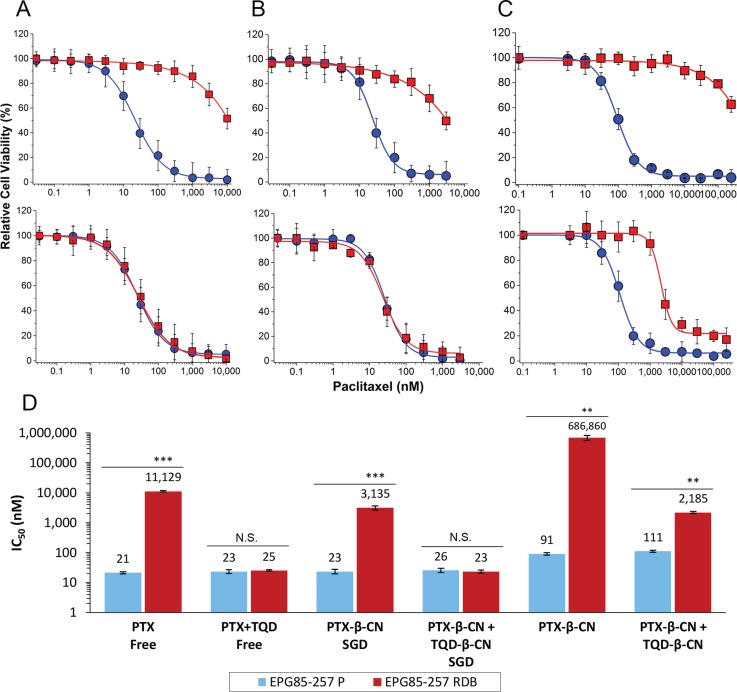
Cytotoxicity assay The relative cell viability as a function of PTX concentration of: (**A**) free (untreated) drugs, (**B**) drug-β-CM following SGD and (**C**) drug-β-CM before SGD was examined using EPG85-257P, parental cell line (●) and EPG85-257RDB multidrug resistant subline (■) in the presence (lower panel) or absence (upper panel) of 0.8 μM TQD. Values presented are means ± SE. Lines represent sigmoidal model curve fits to Equation (6). (**D**) IC_50_ values generated from the fitted dose-response curves; N.S. indicates a non-significant difference, **indicates *P* < 0.001 and ***indicates *P* < 0.0001 as determined by student's *t*-test.

The cytotoxic activity of free PTX was compared with that of PTX- β-CM (6:1 PTX:β-CN molar ratio, before and after SGD) in the presence or absence of 0.8 μM TQD, either free or in β-CM (4:1 TQD:β-CN molar ratio, before and after SGD, respectively). The fraction of surviving cells (relative to untreated controls) vs. PTX concentration is presented in Figure 7A, 7C and the IC_50_ values derived from a sigmoidal fitting of the dose-response curves using Equation (6) are shown in Figure 7D. Cells exposed to a 1 h pulse of SFM containing 1 mg/ml pure undigested β-CN (20% PBS), complete growth medium containing digested β-CN, 0.8 μM free TQD, and samples containing 0.1% DMSO (v/v) in complete growth medium or in SFM were tested as controls. No significant cytotoxicity was observed with these controls (Supplementary Figure S2A). Moreover, cytotoxicity to parental EPG85-257P cells, following a 1 h pulse of free PTX in complete growth medium was compared with free PTX in SFM parental EPG85-257P cells; no substantial cytotoxicity was observed in the presence of SFM (Supplementary Figure S2B). A 530-fold resistance to free PTX was observed in P-gp-overexpressing MDR EPG85-257RDB cells, compared to their parental cells (Figure 7A, 7D) which were devoid of P-gp (Figure 6). The mean IC_50_ values obtained for EPG85-257RDB and EPG85-257P cells were 11,129 ± 598 and 21 ± 2 nM, respectively. This resistance was fully reversed by free TQD (0.8 μM). These results also demonstrate that encapsulation and gastric digestion did not interfere with the cytotoxic activity of the drugs and even improved it in the case of the chemosensitizer as the IC_50_ value for the parental cell line (23 ± 4 nM) was not significantly different (*P* = 0.5944) from that of free PTX, and the IC_50_ value of the MDR subline (3,135 ± 499 nM) was decreased 3.5-fold (Figure 7B, 7D).

The cytotoxic activity of undigested PTX-β-CM with and without undigested TQD-β-CM was determined in SFM. The undigested drug-loaded micelles were tested to evaluate the quality of drug entrapment (or, inversely, the leakiness of the vehicle) prior to gastric digestion. Undigested PTX-β-CM exhibited significantly lower cytotoxicity than free PTX or digested PTX-β-CM, demonstrating an important advantage of encapsulated drug over free drug. The IC_50_ values were 4.3-fold and 4.0-fold higher for the parental cells (91 ± 9 nM) and 62-fold and 219-fold higher for the MDR subline (686,860 ± 134,046 nM), respectively (Figure 7C, 7D). This indicates that the loading of the chemotherapeutic drug was effective but the encapsulation efficiency was lower, ~60% as seen in our results of the calculated encapsulation efficiency (Figure 2B), due to an equilibrium between the drug within the micelles and the excess drug, causing the cytotoxic effect. Expectedly, the cytotoxicity of PTX-β-CM with and without undigested TQD-β-CM towards EPG85-257P cells was not significantly different (*P* = 0.2410). Furthermore, the addition of undigested TQD-β-CM did not achieve a full MDR reversal, even though the IC_50_ value decreased 314-fold, and was similar to that of PTX-β-CM following SGD (*P* = 0.3720).

## DISCUSSION

Based on our previous studies [3, 19–21] which introduced the potential of β-CM as nanovehicles for oral delivery and target-activated release of hydrophobic drug cargo in the stomach, we herein devised a novel nanosystem comprising a potent chemotherapeutic drug (PTX) and a potent chemosensitizer (TQD), individually encapsulated within β-CM, and assessed its *in vitro* efficacy and drug resistance reversal potential using paired MDR human gastric carcinoma cells. Both PTX and TQD displayed good affinity for β-CN, which efficiently entrapped, solubilized and stabilized them colloidally, and prevented them from aggregating and crystallizing (below its maximal loading capacity), as evidenced by several complementary methods, including visual observation, absorbance spectra, fluorescence spectroscopy, light microscopy, dynamic light scattering and zeta potential. These results were in line with previous studies that investigated the binding of lipophilic molecules to β-CN, including vitamin D_3_ [37], vitamin A [38], sucrose esters [39], celecoxib [40] and curcumin [41], suggesting that hydrophobic interactions are predominant in the entrapment of hydrophobic compounds within β-CM.

Oral administration of PTX was recently examined using different vehicles, including glycyrrhizic acid micelles [42], pluronic-based micelles [43, 44], chitosan-based nanovehicles [45, 46] and lipid-based nanoparticles (NPs) [45, 47]. However, none of these vehicles exhibited target-activated release in the stomach. Following a comprehensive literature survey we conclude that neither a carrier for oral delivery of TQD, nor for oral delivery of the combination of PTX and TQD have yet been investigated.

The estimated maximal drug loading capacity was ~20:1 (mol:mol) drug:β-CN for PTX, i.e. 712 mg/g protein, and 5:1 (mol:mol) for TQD, which is 135 mg/g protein. The result for TQD was in agreement with the maximal loading capacity (L_max_, 136 mg/g protein) calculated from the asymptote of the saturation curve (Equation (5)) fitted to the plot in Figure 2A. For PTX, however, the calculated L_max_ (175 mg/g protein) differed from the qualitatively estimated value. This difference may be due to the formation of sub-micrometer crystals of the excess drug, above the L_max_, which are below the resolution limit of the optical microscope, but which sediment upon centrifugation. Since the sedimentation method is more quantitative and reliable, we conclude that L_max_ was ~175 mg/g protein. The L_max_ values we found for PTX were similar, while those for TQD were higher than those reported in previous studies using different nano-delivery systems designed to co-deliver PTX and TQD (each encapsulated individually, and intended for IV administration). A study using poly (D, L-lactide-co-glycolide) NPs [48] reported 17.6% loading capacity (i.e. about 210 mg PTX per gr polymer). They further reported 2.6% TQD loading (i.e. about 27 mg TQD per gr polymer), showing that β-CN displayed a 5-fold higher L_max_ for TQD compared to the PLGL NPs.

Our *in vitro* cytotoxicity results demonstrated that TQD achieved a complete MDR reversal in the human MDR gastric carcinoma cell line EPG85-257RDB. These results confirmed that following SGD, the drugs were properly released and their pharmacologic activity was fully retained. Sustaining drug activity after SGD was consistent with our previous findings for PTX-β-CM with a different cancer cell line [21], suggesting that this drug delivery system is suitable for oral delivery and gastric release of hydrophobic drug combinations for treatment of gastric disorders. Moreover, our current findings revealed that encapsulation followed by SGD enhanced TQD activity ~ 4-fold, presumably due to the suppression of drug crystallization by β-CN. Another important observation was that the encapsulated (and undigested) PTX-β-CM showed significantly lower cytotoxicity than the free or released drug (particularly for the MDR cells). The slight cytotoxicity indicated a loading efficiency < 100%, enabling the excess free drug to exert its activity. To overcome this problem, a lower drug:protein ratio may be used. Alternatively, the use of additional coating layers [45, 49, 50] or protein crosslinking [51, 52] may enhance drug encapsulation efficiency and increase protection against untoward toxicity to the upper gastro-intestinal tract. Previous studies testing similar improvements with different delivery systems exhibited reduced toxicity [50] and improved survival [45]. Target-activated release of chemotherapeutics in the stomach might provoke intestinal toxicity following gastric passage. In order to obtain a maximal therapeutic index, we plan to develop and optimize β-CN-based nanovehicles loaded with the minimal drug dose needed for efficacious local gastric treatment and minimal residual intestinal toxicity, by performing dedicated *in vivo* studies with mouse models harboring human gastric cancer xenografts.

Combinations of PTX and TQD, simultaneously incorporated in a carrier, have been previously evaluated with different delivery systems designed for IV co-administration [48, 53]. This enhanced therapeutic efficacy of the co-administered agents is attributable to increased accumulation of PTX in drug-resistant tumor cells [34, 48, 53]. Oral delivery is clearly desirable over IV, which requires hospitalization, and is hence costly and dangerous for immunocompromised cancer patients.

In conclusion, our findings demonstrate that β-CM exhibit efficient drug encapsulation, solubilization and suppression of crystallization, and high loading capacities of PTX and TQD. β-CM show great promise as an oral nano-delivery platform for gastric target-activated release of co-administered synergistic drug-chemosensitizer combinations, for effectively overcoming MDR in gastric cancer. This novel nanosystem could be applied for the treatment of various gastric disorders and tailoring individual drug combinations in personalized medicine.

## MATERIALS AND METHODS

### Drugs encapsulation in β-CM

Stock solutions of 50 mM PTX (Sigma-Aldrich Ltd., Rehovot, Israel) and 20 mM TQD (MedKoo Bioscience, Chapel Hill, NC, USA) were prepared in dimethyl sulfoxide (DMSO). β-CN stock solutions were prepared by dissolving β-CN (Sigma-Aldrich Ltd., Rehovot, Israel; C6905; ≥ 98% purity) in phosphate-buffered saline (PBS) at pH 7.0 and 0.1 M ionic strength [19]. Drug entrapment in β-CM was performed as previously described [19]. In all samples, β-CN concentration was above the critical micellization concentration of pure β-CN [54]. The volume percentage of DMSO in PBS did not exceed 2.5%.

### Binding studies by spectrofluorimetry

Fluorescence quenching of tryptophan (Trp) 143, located in the hydrophobic domain of β-CN [37, 39], was used to determine the binding affinities of PTX and TQD to β-CN. Trp fluorescence was determined using excitation of 270 nm and recording emission from 300 to 450 nm, with 1 nm slit widths using a Fluorolog 3–22 spectrofluorometer (Horiba, Jobin Yvon, Longjumeau, France). β-CN fluorescence was monitored at a constant protein concentration of 1 mg/ml and either PTX or TQD at increasing drug:β-CN molar ratios. Samples were prepared in PBS containing 2.5% (v/v) DMSO. Measurements were performed in duplicates at 23°C, and the average value and SE were calculated.

Stern-Volmer plots for dynamic quenching were fitted according to Equation (1):

$$\frac{F_{0}}{F}=1+K_{SV}\left[Q\right]\;$$ (1)

Non-linear upward-curving of the Stern-Volmer plots were observed indicating that both static and dynamic quenching mechanisms take place during the binding. Hence, association constant calculations were made using the modified Stern-Volmer equation [55]:

$$\frac{F_{0}}{F}=\left(1+K_{SV}\left[D\right]\right)exp^{\left(V\left[D\right]\right)}\;$$ (2)

Where F and F_0_ are β-CN fluorescence intensity in the presence and absence of the drug, respectively; [D] is the drug concentration, V is the static quenching constant and K_SV_ is the Stern-Volmer overall quenching constant. Data fitting was calculated using OriginPro 9.0 software (OriginLab Corporation, Northampton, MA, USA).

### Drug loading analysis

To quantify the amount of drug loaded into the β-CM, samples of 1 mg/ml protein containing either PTX or TQD at rising drug:β-CN molar ratios were prepared in PBS containing 1.0% (v/v) DMSO. The samples were centrifuged at 10,000 × g at 4°C for 20 min. The supernatant was collected and the pellets were dissolved in DMSO and equilibrated at room temperature (RT) for 1 h. The drug concentration was determined using an Ultrospec 3000 spectrophotometer. Analysis was performed using suitable linear calibration curves. Results are presented as means ± SE of two independent experiments, each performed in duplicates.

Analysis of the loading capacity (L) and encapsulation efficiency (E) of each of the drugs in β-CM was performed using Eqs. (3) and (4) [56]:

$$\begin{array}{cc} L\;\left(\frac{mg\;drug}{g\;\beta \;-\;CN}\right)\;=\;\frac{W_{ED}}{W_{\beta -CN}}\;=\;\frac{W_{TD}-W_{SD}}{W_{\beta -CN}} & 0 \end{array}$$ (3)

$$\begin{array}{c} E\;\left(\%\right)\;=\;\frac{W_{ED}}{W_{TD}}\;100 \end{array}$$ (4)

Where W_ED_ is the amount of encapsulated drug, W_TD_ is the total amount of drug in the sample, W_SD_ is the amount of unencapsulated drug which sedimented and was quantified in the centrifugation pellet and W_β-CN_ is the amount of the protein in the sample. Loading capacity data analysis, used to estimate L_max_, was performed by employing a nonlinear curve fitting with OriginPro 9.0, according to the Langmuir equation:

$$L=\frac{L_{max}K\;\left[D\right]}{1+K\;\left[D\right]}\;$$ (5)

Where L_max_ is the maximal loading capacity (saturation asymptote), K is the Langmuir sorption constant and [D] is the drug concentration.

### Binding stoichiometry evaluation by light microscopy

The binding stoichiometry, morphology and crystal formation of the drugs with and without β-CN were studied by light microscopy. The pictures were obtained using an Olympus DP71 digital camera connected to an Olympus BX51 light microscope operated with Nomarski differential interference contrast (DIC) and polarized light optics (x20 magnification, 24°C). Samples of 1 mg/ml β-CN, 667 and 833 μM pure PTX, 208 and 333 μM pure TQD, and of β-CM with each of these drugs at the following molar ratio of drug:β-CN, respectively: 16:1, 20:1 5:1, and 8:1, were prepared in PBS containing 1.67% (v/v) DMSO. Approximately 10 similar images of each of the samples were collected in three independent experiments.

### Particles size distribution analysis by dynamic light scattering (DLS)

Volume-weighted particle size distributions of the micellar complexes at different drug:β-CN molar ratios were studied using a DLS analyzer, NICOMP^™^ 380 (Particle Sizing System (PSS), Inc., Santa Barbara, CA, USA). Scattered light intensity was detected by an Avalanche photo diode detector at a fixed angle of 90°. The laser wavelength was 658 nm. Nicomp, mono-, bi-, or tri-modal distributions were calculated from the scattered light intensity fluctuations [3]. Samples were prepared in PBS with 2.0% (v/v) DMSO. Measurements were made in triplicates, and mean values and SE were calculated.

### Zeta potential analysis

Electrophoretic mobility was determined using a Zetasizer Nano-ZS (ZEN3600) instrument (Malvern Instruments Ltd., Worcestershire, UK). Zeta potential was derived based on the Smoluchowski model (Applicable when the ratio of particle size (a) to Debye length (κ^1^) is >> 1 [57]. Here κ^1^≈2.15 nm and particle sizes (a) were 365 nm and 265 nm for PTX-β-CM and TQD-β-CM, thus κa values were 170 and 123, respectively; i.e. κa >> 1). Samples of 1 mg/ml (42 μM) β-CN, 250 μM pure PTX, 167 μM pure TQD, and of β-CM with each of these drugs at 6:1 and 4:1 drug:β-CN molar ratio, respectively, were prepared in PBS containing 0.5% (v/v) DMSO. Measurements were made in triplicates at 25°C, the average value and SE were calculated.

### Tissue culture

Human gastric carcinoma EPG85-257P cells and their MDR subline EPG85-257RDB (overexpressing P-gp), were generously provided by Prof. H. Lage (Charité - Medicine University, Berlin, Germany). Cells were cultured in Leibovitz L-15 medium, supplemented with 6.25 mg/l fetuin, 2.5 mg/l transferrin, 0.5 g/l D-glucose (Sigma-Aldrich Ltd., Rehovot, Israel), 10% fetal calf serum, 1% minimal essential vitamins, 1 mg/l glutamine, 80 IE/l insulin, 100 μg/ml penicillin, 100 units/ml streptomycin (Biological Industries, Kibbutz Beit-HaEmek, Israel) and 1.12 g/l NaHCO_3_ (Frutarom, Haifa, Israel). The growth medium of the MDR subline was supplemented with 2.5 μg/ml (4.4 μM) daunorubicin (Sigma-Aldrich Ltd., Rehovot, Israel). MDR cells were grown in drug-free medium for a week prior to each experiment.

### Quantification of P-gp expression by Western blot analysis

P-gp expression was determined by Western blot analysis. Membrane proteins were isolated using lysis buffer containing 50 mM Tris pH 7.5, 50 mM β-mercaptoethanol, 0.5% Triton X-100, protease inhibitor cocktail (Roche Diagnostics, Mannheim, Germany), 1 mM EGTA and 1 mM EDTA. The extract was incubated on ice for 1 h, centrifuged at 15,000 × g at 4°C for 30 min and the protein containing supernatant was collected. Protein concentration was determined using the Bradford assay (Bio-Rad, Hercules, CA, USA). Protein samples (10 μg) were resolved by electrophoresis on 7% polyacrylamide gel containing SDS, electroblotted onto a nitrocellulose membrane and blocked for 1 h at RT in blocking buffer (Tris-buffered saline (TBS); 150 mM NaCl, 20 mM Tris-base at pH 8.0, containing 1% skim milk and 0.5% Tween 20). The blots were incubated with a mouse anti-P-gp monoclonal antibody, JSB-1 (1:100 dilution, overnight at 4°C; kindly provided by Prof. R. J. Scheper and Dr. G. L. Scheffer, VU University Medical Center, Amsterdam, The Netherlands). The blots were then rinsed three times for 10 min each in washing buffer (TBS containing 0.5% Tween 20) at RT and reacted with horseradish peroxidase (HRP)-conjugated goat anti-mouse IgG (1:10,000 dilution, 1 h at RT; Jackson ImmunoResearch Laboratories, West Grove, PA, USA). Following three 10 min washes at RT, detection using an enhanced chemiluminescence (Biological Industries, Kibbutz Beit-HaEmek, Israel) was recorded using an imaging analysis system (ImageQuant LAS 4000, GE Healthcare). Actual protein loading was confirmed using a rabbit polyclonal antibody against the α-subunit of Na^+^/K^+^ ATPase, KETTY (1:3,000 dilution; kindly provided by Prof. S. J. D. Karlish, Weizmann Institute of Science, Rehovot, Israel) and detected with HRP-conjugated goat anti-rabbit IgG (1:15,000 dilution; Jackson ImmunoResearch Laboratories, West Grove, PA, USA).

### Simulated gastric digestion (SGD)

SGD for the cytotoxicity experiments was performed using the method described and at the optimal conditions determined previously [21]. The drug concentration was determined using an Ultrospec 3000 spectrophotometer.

### Cytotoxicity assays

To determine cytotoxicity of PTX in the presence and absence of 0.8 μM TQD, the DMSO solutions of the free drugs and the drugs released by SGD were diluted in complete growth medium. Cytotoxicity of undigested PTX-β-CM was studied in serum-free medium (SFM) due to competitive binding of hydrophobic drugs to human serum albumin as previously described [21]. Our system is designed for oral delivery, hence, the β-CM are not expected to come in contact with serum albumin. Undigested β-CM were prepared by adding a constant volume of different concentrations of PTX or TQD in DMSO to a 5 mg/ml β-CN solution in PBS while continuously stirring, to obtain a range of PTX concentrations of: 15–1,250 μM and 4 μM TQD. The samples were diluted 5-fold in SFM and equilibrated overnight at 4°C before mixing them for the combination experiments.

Cells were seeded at 2.5 × 10^3^ cells/well in 96-well plates (100 μl/well). Following a 24 h incubation, cells were exposed to the different systems at increasing PTX concentrations (0.1% (v/v) DMSO) for 1 h (to simulate an average residence time in the stomach, which ranges from 30 min to 4 h after food ingestion [58]), followed by three washes with complete growth medium or PBS (for the undigested systems) to remove the excess drug. After an additional 48 h of incubation (to allow the drug absorbed to act on the cells), cell viability was determined using an XTT-based cell proliferation assay (Biological Industries, Kibbutz Beit-HaEmek, Israel) [21]. Results shown are means ± SE obtained from three independent experiments, each performed in triplicates.

Cytotoxicity data were analyzed using a nonlinear curve fitting of a sigmoidal model (Hill1) with OriginPro 9.0 for dose-response curve according to Equation (6) [21]:

$$P=P_{\infty }+\left(P_{0}-P_{\infty }\right)\left(\frac{{\left[D\right]}^{n}}{IC_{50}^{n}+{\left[D\right]}^{n}}\right)\;$$ (6)

Where P represents the percentage of live cells at each drug concentration, P_0_ represents the maximal percent of surviving cells in the absence of drug (100% = control), P_∞_ represents the minimal percent of live cells at infinite drug concentration (maximal response value characterized by zero viability of cells), and [D] represents the drug concentration; IC_50_ is the drug concentration exerting 50% inhibition of cell growth and n is the Hill slope parameter for the abruptness of the dose-response curve. The statistical analysis of variance of the calculated IC_50_ values was determined by an unpaired student's *t*-test. A *P*-value lower than 0.05 was considered as indicating statistical significance.

## SUPPLEMENTARY MATERIALS FIGURES



## References

[1] DeSantis CE, Lin CC, Mariotto AB, Siegel RL, Stein KD, Kramer JL, Alteri R, Robbins AS, Jemal A (2014). Cancer treatment and survivorship statistics, 2014. CA Cancer J Clin.

[2] Nagini S (2012). Carcinoma of the stomach: A review of epidemiology, pathogenesis, molecular genetics and chemoprevention. World J Gastrointest Oncol.

[3] Shapira A, Assaraf YG, Livney YD (2010). Beta-casein nanovehicles for oral delivery of chemotherapeutic drugs. Nanomedicine.

[4] Liu G, Franssen E, Fitch MI, Warner E (1997). Patient preferences for oral versus intravenous palliative chemotherapy. J Clin Oncol.

[5] Ashour HM, el-Sharif A (2007). Microbial spectrum and antibiotic susceptibility profile of gram-positive aerobic bacteria isolated from cancer patients. J Clin Oncol.

[6] Shapira A, Livney YD, Broxterman HJ, Assaraf YG (2011). Nanomedicine for targeted cancer therapy: Towards the overcoming of drug resistance. Drug Resist Updates.

[7] Assaraf YG (2007). Molecular basis of antifolate resistance. Cancer Metastasis Rev.

[8] Gonen N, Assaraf YG (2012). Antifolates in cancer therapy: structure, activity and mechanisms of drug resistance. Drug Resist Updates.

[9] Livney YD, Assaraf YG (2013). Rationally designed nanovehicles to overcome cancer chemoresistance. Adv Drug Deliv Rev.

[10] Zhitomirsky B, Assaraf YG (2016). Lysosomes as mediators of drug resistance in cancer. Drug Resist Updates.

[11] Szakacs G, Paterson JK, Ludwig JA, Booth-Genthe C, Gottesman MM (2006). Targeting multidrug resistance in cancer. Nat Rev Drug Discov.

[12] Cordon-Cardo C, O'Brien JP, Boccia J, Casals D, Bertino JR, Melamed MR (1990). Expression of the multidrug resistance gene product (P-glycoprotein) in human normal and tumor tissues. J Histochem Cytochem.

[13] Shukla S, Ohnuma S, Ambudkar SV (2011). Improving cancer chemotherapy with modulators of ABC drug transporters. Curr Drug Targets.

[14] Mei L, Zhang Z, Zhao L, Huang L, Yang XL, Tang J, Feng SS (2013). Pharmaceutical nanotechnology for oral delivery of anticancer drugs. Adv Drug Deliv Rev.

[15] Huynh NT, Passirani C, Saulnier P, Benoit JP (2009). Lipid nanocapsules: A new platform for nanomedicine. Int J Pharm.

[16] Thanki K, Gangwal RP, Sangamwar AT, Jain S (2013). Oral delivery of anticancer drugs: challenges and opportunities. J Control Release.

[17] Livney YD, Schwan AL, Dalgleish DG (2004). A Study of β-Casein Tertiary Structure by Intramolecular Crosslinking and Mass Spectrometry. J Dairy Sci.

[18] Mikheeva LM, Grinberg NV, Grinberg VY, Khokhlov AR, de Kruif CG (2003). Thermodynamics of Micellization of Bovine β-Casein Studied by High-Sensitivity Differential Scanning Calorimetry. Langmuir.

[19] Shapira A, Assaraf YG, Epstein D, Livney YD (2010). Beta-casein Nanoparticles as an Oral Delivery System for Chemotherapeutic Drugs: Impact of Drug Structure and Properties on Co-assembly. Pharm Res.

[20] Shapira A, Markman G, Assaraf YG, Livney YD (2010). β-casein–based nanovehicles for oral delivery of chemotherapeutic drugs: drug-protein interactions and mitoxantrone loading capacity. Nanomedicine.

[21] Shapira A, Davidson I, Avni N, Assaraf YG, Livney YD (2012). β-Casein nanoparticle-based oral drug delivery system for potential treatment of gastric carcinoma: Stability, target-activated release and cytotoxicity. Eur J Pharm Biopharm.

[22] Livney YD (2010). Milk proteins as vehicles for bioactives. Curr Opin Colloid Interface Sci.

[23] Semo E, Kesselman E, Danino D, Livney YD (2007). Casein micelle as a natural nano-capsular vehicle for nutraceuticals. Food Hydrocol.

[24] Haham M, Ish-Shalom S, Nodelman M, Duek I, Segal E, Kustanovich M, Livney YD (2012). Stability and bioavailability of vitamin D nanoencapsulated in casein micelles. Food Funct.

[25] Benede S, Lopez-Exposito I, Gimenez G, Grishina G, Bardina L, Sampson HA, Molina E, Lopez-Fandino R (2014). *In vitro* digestibility of bovine beta-casein with simulated and human oral and gastrointestinal fluids. Identification and IgE-reactivity of the resultant peptides. Food Chem.

[26] Journo-Gershfeld G, Kapp D, Shamay Y, Kopeček J, David A (2012). Hyaluronan Oligomers-HPMA Copolymer Conjugates for Targeting Paclitaxel to CD44-Overexpressing Ovarian Carcinoma. Pharm Res.

[27] Iyer SS, Gao S, Zhang ZP, Kellogg GE, Karnes HT (2005). A molecular model to explain paclitaxel and docetaxel sensitivity changes through adduct formation with primary amines in electrospray ionization mass spectrometry. Rapid Commun Mass Spectrom.

[28] Montagner IM, Banzato A, Zuccolotto G, Renier D, Campisi M, Bassi P, Zanovello P, Rosato A (2013). Paclitaxel-hyaluronan hydrosoluble bioconjugate: Mechanism of action in human bladder cancer cell lines. Urol Oncol.

[29] Egusquiaguirre SP, Igartua M, Hernández RM, Pedraz JL (2012). Nanoparticle delivery systems for cancer therapy: advances in clinical and preclinical research. Clin Transl Oncol.

[30] Egger M, Li X, Müller C, Bernhardt G, Buschauer A, König B (2007). Tariquidar Analogues: Synthesis by CuI-Catalysed N/O–Aryl Coupling and Inhibitory Activity against the ABCB1 Transporter. Eur J Org Chem.

[31] Fox E, Bates SE (2007). Tariquidar (XR9576): a P-glycoprotein drug efflux pump inhibitor. Expert Rev Anticancer Ther.

[32] Hendrikx JJ, Lagas JS, Wagenaar E, Rosing H, Schellens JH, Beijnen JH, Schinkel AH (2014). Oral co-administration of elacridar and ritonavir enhances plasma levels of oral paclitaxel and docetaxel without affecting relative brain accumulation. Br J Cancer.

[33] Sarisozen C, Vural I, Levchenko T, Hincal AA, Torchilin VP (2012). PEG-PE-based micelles co-loaded with paclitaxel and cyclosporine A or loaded with paclitaxel and targeted by anticancer antibody overcome drug resistance in cancer cells. Drug deliv.

[34] Wang F, Zhang D, Zhang Q, Chen Y, Zheng D, Hao L, Duan C, Jia L, Liu G, Liu Y (2011). Synergistic effect of folate-mediated targeting and verapamil-mediated P-gp inhibition with paclitaxel -polymer micelles to overcome multi-drug resistance. Biomaterials.

[35] Lakowicz JR (2006). Principles of Fluorescence Spectroscopy.

[36] Ferenc M, Pedziwiatr-Werbicka E, Nowak K, Klajnert B, Majoral J-P, Bryszewska M (2013). Phosphorus Dendrimers as Carriers of siRNA—Characterisation of Dendriplexes. Molecules.

[37] Forrest SA, Yada RY, Rousseau D (2005). Interactions of Vitamin D3 with Bovine β-Lactoglobulin A and β-Casein. J Agric Food Chem.

[38] Lietaer E, Poiffait A, Adrian J (1991). Interaction between casein and vitamin A. Lebensm-Wiss Technol.

[39] Clark DC, Wilde PJ, Wilson DR, Wustneck R (1992). The interaction of sucrose esters with β-lactoglobulin and β-casein from bovine milk. Food Hydrocol.

[40] Bachar M, Mandelbaum A, Portnaya I, Perlstein H, Even-Chen S, Barenholz Y, Danino D (2012). Development and characterization of a novel drug nanocarrier for oral delivery, based on self-assembled β-casein micelles. J Control Release.

[41] Esmaili M, Ghaffari SM, Moosavi-Movahedi Z, Atri MS, Sharifizadeh A, Farhadi M, Yousefi R, Chobert J-M, Haertlé T, Moosavi-Movahedi AA (2011). Beta casein-micelle as a nano vehicle for solubility enhancement of curcumin; food industry application. Lebensm-Wiss Technol.

[42] Yang FH, Zhang Q, Liang QY, Wang SQ, Zhao BX, Wang YT, Cai Y, Li GF (2015). Bioavailability Enhancement of Paclitaxel via a Novel Oral Drug Delivery System: Paclitaxel-Loaded Glycyrrhizic Acid Micelles. Molecules.

[43] Zhao YL, Li YL, Ge JJ, Li N, Li LB (2014). Pluronic-poly (acrylic acid)-cysteine/Pluronic L121 mixed micelles improve the oral bioavailability of paclitaxel. Drug Dev Ind Pharm.

[44] Xu W, Fan X, Zhao Y, Li L (2015). Cysteine modified and bile salt based micelles: Preparation and application as an oral delivery system for paclitaxel. Colloids Surf, B.

[45] Joshi N, Saha R, Shanmugam T, Balakrishnan B, More P, Banerjee R (2013). Carboxymethyl-Chitosan-Tethered Lipid Vesicles: Hybrid Nanoblanket for Oral Delivery of Paclitaxel. Biomacromol.

[46] Battogtokh G, Ko Y (2014). Self-assembled Chitosan-Ceramide Nanoparticle for Enhanced Oral Delivery of Paclitaxel. Pharm Res.

[47] Pandita D, Ahuja A, Lather V, Benjamin B, Dutta T, Velpandian T, Khar R (2011). Development of Lipid-Based Nanoparticles for Enhancing the Oral Bioavailability of Paclitaxel. AAPS Pharm Sci Tech.

[48] Patil Y, Sadhukha T, Ma L, Panyam J (2009). Nanoparticle-mediated simultaneous and targeted delivery of paclitaxel and tariquidar overcomes tumor drug resistance. J Control Release.

[49] Jain S, Kumar D, Swarnakar NK, Thanki K (2012). Polyelectrolyte stabilized multilayered liposomes for oral delivery of paclitaxel. Biomaterials.

[50] Jain S, Patil SR, Swarnakar NK, Agrawal AK (2012). Oral Delivery of Doxorubicin Using Novel Polyelectrolyte-Stabilized Liposomes (Layersomes). Mol Pharm.

[51] Zambito Y, Felice F, Fabiano A, Di Stefano R, Di Colo G (2013). Mucoadhesive nanoparticles made of thiolated quaternary chitosan crosslinked with hyaluronan. Carbohydr Polym.

[52] Puga AM, Lima AC, Mano JF, Concheiro A, Alvarez-Lorenzo C (2013). Pectin-coated chitosan microgels crosslinked on superhydrophobic surfaces for 5-fluorouracil encapsulation. Carbohydr Polym.

[53] Patel NR, Rathi A, Mongayt D, Torchilin VP (2011). Reversal of multidrug resistance by co-delivery of tariquidar (XR9576) and paclitaxel using long-circulating liposomes. Int J Pharm.

[54] Portnaya I, Cogan U, Livney YD, Ramon O, Shimoni K, Rosenberg M, Danino D (2006). Micellization of Bovine Beta Casein Studied by Isothermal Titration Microcalorimetry and Cryogenic Transmission Electron Microscopy. J Agric Food Chem.

[55] Eftink MR, Ghiron CA (1976). Exposure of Tryptophanyl Residues in Proteins - Quantitative-Determination by Fluorescence Quenching Studies. Biochem.

[56] Penalva R, Esparza I, Agüeros M, Gonzalez-Navarro CJ, Gonzalez-Ferrero C, Irache JM (2015). Casein nanoparticles as carriers for the oral delivery of folic acid. Food Hydrocol.

[57] Delgado AV, Gonzalez-Caballero E, Hunter RJ, Koopal LK, Lyklema J (2005). Measurement and interpretation of electrokinetic phenomena - (IUPAC technical report). Pure Appl Chem.

[58] Washington N, Washington C, Wilson CG (2001). Physiological Pharmaceutics. Biological Barriers to Drug Absorption.

