# Supplementary material for: β-casein nanovehicles for oral delivery of chemotherapeutic drug combinations overcoming P-glycoprotein-mediated multidrug resistance in human gastric cancer cells
Source: Oncotarget. 2016 Mar 10;7(17):23322–34. doi: 10.18632/oncotarget.8019 (PMC5029629; doi:10.18632/oncotarget.8019)
Supplement: Supplementary file 1 [file oncotarget-07-23322-s001.pdf]

β-casein nanovehicles for oral delivery of chemotherapeutic drug combinations overcoming P-glycoprotein-mediated multidrug resistance in human gastric cancer cells

Supplementary Materials

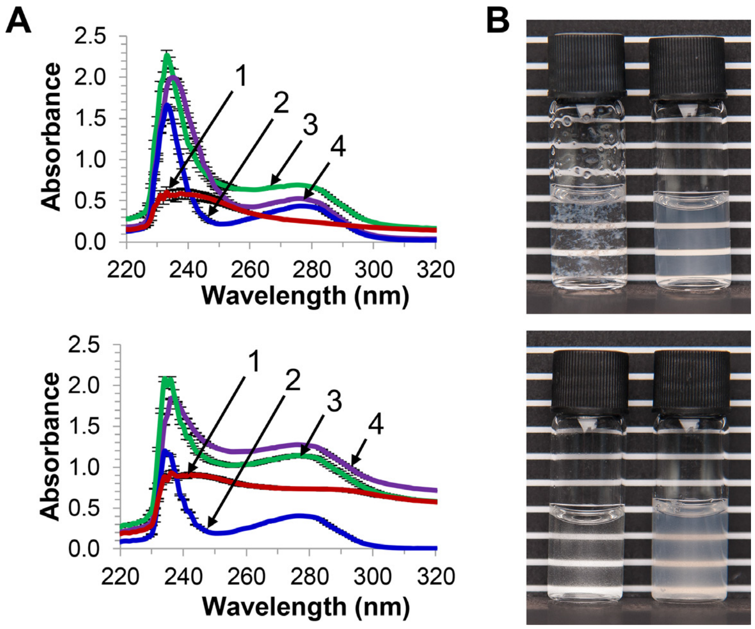

Supplementary Figure S1: Drug-β-CN interaction studies. (**A**) Absorbance spectra of (1) 42 µM pure PTX (upper panel) or TQD (lower panel), (2) 1 mg/ml (42 µM) pure β-CN, (3) mathematical summation plot of the former two spectra vs. (4) their complex (1:1 drug:β-CN molar ratio). Error bars represent SE. (**B**) Photographs of 167 µM pure PTX and TQD in PBS (left) vs. each of the drugs, at the same concentration, in 1 mg/ml β-CN (4:1 drug:β-CN molar ratio) (right).

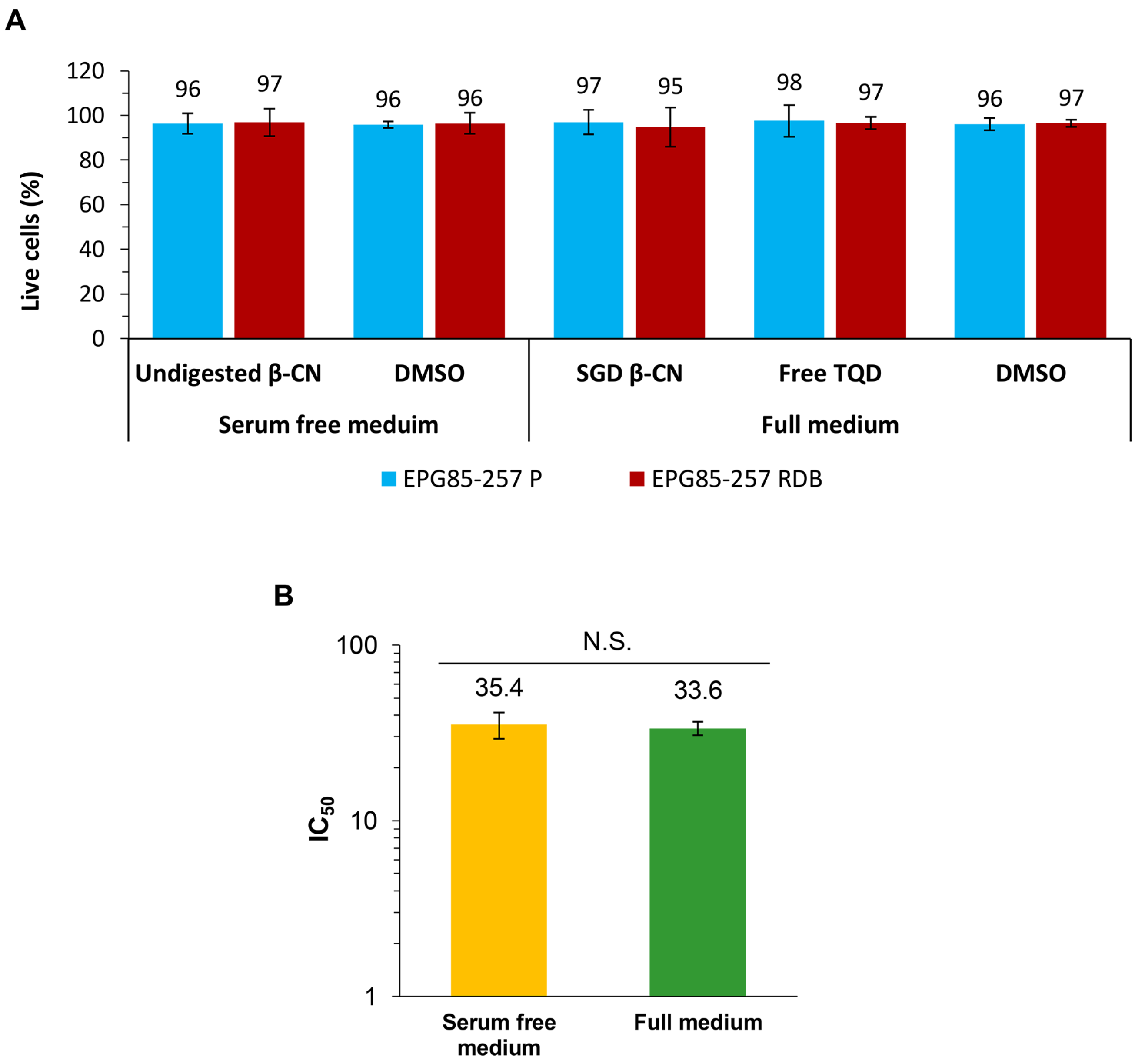

Supplementary Figure S2: Cytotoxicity assay controls. (**A**) Percentage of live cells in the different control solutions: 1 mg/ml pure undigested β-CN in SFM (containing up to 20% PBS), digested β-CN in full growth medium, 0.8 μM free TQD in full growth medium and 0.1% (v/v) DMSO in full growth medium and SFM. (**B**) IC_{50} values of free PTX in full growth medium in comparison with free PTX in SFM. N.S. indicates a non-significant difference, as determined by student's *t*-test.

SUPPLEMENTARY MATERIALS AND METHODS

Drug-β-CN interactions revealed by UV-Vis absorbance spectra analysis

Drug-β-CN interactions were studied by UV-Vis absorbance spectra analysis of 1 mg/ml (42 μM) β-CN, 42 μM PTX or TQD and the β-CM with each of the drugs at 1:1 drug:β-CN molar ratio. Samples were prepared in PBS containing 0.6% (v/v) DMSO and their analyses were performed against a suitable blank. Spectra were collected using an Ultrospec 3000 spectrophotometer (GE Healthcare, Waukesha, WI, USA). Measurements were made in duplicates at 24°C, and the average value and standard error (SE) were calculated.

The absorbance spectra of two components solution is expected to be additive unless an interaction occurs between them [34]. The absorbance spectrum of each of the drugs in the presence of β-CN versus the mathematical summation of the absorbance spectra of the individual components are shown in Supplementary Figure S1A. It is evident that the absorbance curve of PTX-β-CM differs from the summation curve, indicating an association interaction between these two components; the same relationship was observed for TQD-β-CM.

Evaluation of solubilization by visual observation

Samples of 167 μM pure PTX or TQD and of the respective drug at the same concentration in drug:β-CM (1 mg/ml β-CN, at 4:1 molar ratio) were prepared in PBS containing 1.67% (v/v) DMSO. Photographs were taken using a digital SLR camera, Nikon D700, with a Tamaron AF 28–75 mm f/2.8 XR Di LD lens. Images of each sample were obtained in two independent experiments. It is evident from Supplementary Figure S1B that the free drugs form visible and unstable aggregates which tend to precipitate. In contrast, in the presence of β-CN, the drugs were solubilized due to their entrapment within the protein micelles, resulting in a uniform, sediment-free aqueous nano-dispersion.

Controls for the cytotoxicity assay

The percentage of live cells following a 1 h pulse of control samples containing 1 mg/ml pure undigested β-CN in SFM (containing up to 20% PBS), digested β-CN or 0.8 μM free TQD in full growth medium were compared. Cellular viability was determined using a standard XTT-based cytotoxicity assay preformed on EPG85–257P cells and their multidrug resistant EPG85–257RDB subline. Surviving fraction of cells versus PTX concentration is presented in Supplementary Figure S2A. A control examining the effect of SFM on the cells viability was performed. The cytotoxicity of free PTX in full growth medium was compared to that in SFM following a 1 h pulse on EPG85–257P cell line, and the IC_{50} values determined are described in Supplementary Supplementary Figure S2B.
